# The beneficial effects of *Ganoderma lucidum* on cardiovascular and metabolic disease risk

**DOI:** 10.1080/13880209.2021.1969413

**Published:** 2021-08-31

**Authors:** Sze Wa Chan, Brian Tomlinson, Paul Chan, Christopher Wai Kei Lam

**Affiliations:** a School of Health Sciences, Caritas Institute of Higher Education, Hong Kong SAR, China; b Faculty of Medicine, Macau University of Science & Technology, Macau, China; c Division of Cardiovascular Medicine, Department of Internal Medicine, Wan Fang Hospital, Taipei Medical University, Taipei City, Taiwan

**Keywords:** Antihypertensive, antioxidant, dyslipidaemia, hypoglycaemic, Lingzhi, Reishi

## Abstract

**Context:**

Various herbal medicines are thought to be useful in the management of cardiometabolic disease and its risk factors. *Ganoderma lucidum* (Curtis) P. Karst. (Ganodermataceae), also known as Lingzhi, has received considerable attention for various indications, including some related to the prevention and treatment of cardiovascular and metabolic disease by ameliorating major cardiovascular risk factors.

**Objective:**

This review focuses on the major studies of the whole plant, plant extract, and specific active compounds isolated from *G. lucidum* in relation to the main risk factors for cardiometabolic disease.

**Methods:**

References from major databases including PubMed, Web of Science, and Google Scholar were compiled. The search terms used were *Ganoderma lucidum*, Lingzhi, Reishi, cardiovascular, hypoglycaemic, diabetes, dyslipidaemia, antihypertensive, and anti-inflammatory.

**Results:**

A number of *in vitro* studies and *in vivo* animal models have found that *G. lucidum* possesses antioxidative, antihypertensive, hypoglycaemic, lipid-lowering, and anti-inflammatory properties, but the health benefits in clinical trials are inconsistent. Among these potential health benefits, the most compelling evidence thus far is its hypoglycaemic effects in patients with type 2 diabetes or hyperglycaemia.

**Conclusions:**

The inconsistent evidence about the potential health benefits of *G. lucidum* is possibly because of the use of different *Ganoderma* formulations and different study populations. Further large controlled clinical studies are therefore needed to clarify the potential benefits of *G. lucidum* preparations standardised by known active components in the prevention and treatment of cardiometabolic disease.

## Introduction

Cardiovascular disease (CVD) is highly prevalent, with ischaemic heart disease and stroke being the two leading causes of mortality throughout the world (World Health Organization 2021). Metabolic syndrome is characterised by a cluster of conditions including insulin resistance, central obesity, hypertension, dyslipidaemia, and low-grade chronic inflammation (Eckel et al. 2005). Several drug treatments for CVD have been derived from plant sources, such as digoxin and reserpine. Herbal medicines are now becoming more popular, representing a potentially cost-effective class of substances for combating CVD if safe and effective therapies can be identified. The common herbal medicines used in the West include Asian ginseng, astragalus, flaxseed oil, garlic, ginkgo, grape seeds, green tea, hawthorn, milk thistle, and soy (Liperoti et al. 2017). Herbal formulae are widely used in the clinic in China for hypertension, dyslipidaemia, coronary heart disease, and heart failure (Liu and Huang 2016).

*Ganoderma* (Ganodermataceae) is a kind of woody mushroom that can be found all over the world. Individual members of the species are identified according to different characteristics, such as shape and colour (red, black, blue/green, white, yellow, and purple) of the fruiting bodies, host specificity, and geographical origin (Upton 2000; Wachtel-Galor et al. 2011). *Ganoderma lucidum* (Curtis) P. Karst. (Curtis 1781), known as Lingzhi in China and Reishi in Japan, has been used in traditional Chinese medicine (TCM) for over 2000 years for a broad range of indications including improving general health, well-being, and longevity (Bishop et al. 2015; Klupp et al. 2015).

A variety of commercial products from *G. lucidum*, such as powders, dietary supplements, and tea (Wachtel-Galor et al. 2011), are available. They have been shown to possess a range of activities against CVD, including effects on lipids, blood pressure, obesity, diabetes, and antioxidant and radical scavenging properties (Liu and Tie 2019; Meng and Yang 2019; Winska et al. 2019). However, scientific evidence supporting the beneficial medical properties of *G. lucidum* is still inconclusive (Hapuarachchi et al. 2016). Many of the commercial products from *G. lucidum* may not have undergone effective standardisation, so it is difficult to compare results from different studies with different products. Many different herbal supplements or nutraceutical commercial products bearing the names Lingzhi, Reishi, or *Ganoderma*, etc., contain extracts from various parts of *G. lucidum*, often in combination with other herbal components. Ganopoly™ (Encore Health), which is a product containing water-soluble *G. lucidum* polysaccharides, has been used in some animal and clinical studies.

## Methods

In this review, the major studies of the whole plant, plant extract, and specific active compounds isolated from *G. lucidum* in relation to the main risk factors for CVD with particular emphasis on the more recent studies, are summarised. Electronic literature searches were performed using PubMed, Web of Science, and Google Scholar (published from 1961 to 2021). The search terms used were *Ganoderma lucidum*, Lingzhi, Reishi, cardiovascular, hypoglycaemic, diabetes, dyslipidaemia, antihypertensive, and anti-inflammatory. A total of 4224 articles were identified. The bibliographies of all relevant articles thus located were also scanned for further relevant references. S.W.C and B.T. extracted all articles independently based on the relevance, quality, and strength of the studies; only a shortlist of 115 studies or representative findings are discussed below.

### *Active constituents of* G. lucidum

*G. lucidum* is thought to have numerous different biologically active constituents, the main ones being various triterpenes, polysaccharides, and proteins (Ahmad 2018; Ahmad et al. 2013). The pharmacologically active compounds are present in different amounts in various parts of the mushroom such as the fruiting bodies, mycelium and spores.

### Triterpenes

Terpenes are a large and diverse group of naturally occurring compounds derived from the branched C5 carbon skeleton of isoprene. Triterpenes are a subclass of terpenes and are derived from squalene, a C30 hydrocarbon (Abdullah et al. 2012). They can be classified based on the number of cyclic structures making up the compounds. Up to now, more than 150 triterpenes have been identified from the spores, fruiting bodies, and mycelia of *G. lucidum* (Xia et al. 2014; Baby et al. 2015). The methods of extraction of triterpenes usually involve methanol, ethanol, chloroform, ether, acetone, or a mixture of these solvents. The extracts can be further purified by various separation methods such as normal and reverse-phase high-performance liquid chromatography (HPLC) (Chen et al. 1999). The majority of triterpenes identified are ganoderic acids and lucidenic acids; other important triterpenes include ganodermic acids, ganoderals, and ganoderiols (Wachtel-Galor et al. 2011). The strong bitterness of *G. lucidum* originates from the triterpenoid compounds and the bitterness depends on the strain, cultivation conditions and manufacturing processes (Seo et al. 2009). Triterpenoids have been reported to exhibit various biological activities including anti-hypertensive, lipid-lowering, anti-acetylcholinesterase, antioxidant, and anticancer activities, etc. (Abdullah et al. 2012; Chen et al. 2017).

### Polysaccharides and peptidoglycans

*G. lucidum* polysaccharides are macromolecules with a molecular mass of above 500 kDa. Many different polysaccharides, including (1→3), (1→6)-α/β-glucans, α-d-glucans, α-d-mannans, and polysaccharide-protein complexes, have been identified from the spores, fruiting bodies and mycelia of *G. lucidum*. These compounds are reported to have immunomodulatory and anticancer activities (Xu et al. 2011; Kao et al. 2013). Glucose, together with xylose, mannose, galactose, and fucose in different conformations, forms the major component of the polysaccharide molecules. Polysaccharides are the major component by weight among all constituents in the spores. Several of the mushroom polysaccharide compounds have proceeded through Phase I, II, and III clinical trials and have been used in some Asian countries to treat various cancers and other diseases (Wasser 2010). The contents of polysaccharides differ among commercial Lingzhi products (Wachtel-Galor et al. 2011). A polysaccharide-based product extracted from the spores of *G. lucidum* originally named ‘Ji 731 Injection’ was used since 1973 in China for treating myopathy (Zeng et al. 2018). The drug was renamed ‘Ji Sheng Injection’ in 1985 and subsequently ‘Polysaccharidum of *G. lucidum* Karst Injection’ (Lin Bao Duo Tang Zhu She Ye) and is still used for intramuscular injection for various types of immune-mediated muscle diseases. Various bioactive peptidoglycans possessing antiviral (Li et al. 2005) and immunomodulating activities (Zhang et al. 2019), such as ganoderans A, B, and C, have also been isolated from *G. lucidum*.

### Bioactive proteins

Several bioactive proteins from *G. lucidum* have been reported. One of these is a polypeptide called Lingzhi-8 (LZ-8) which consists of 110 amino acids with a molecular mass of 12 kDa. It has an immunoglobulin-like structure and was the first immunomodulatory protein isolated from the mushroom in 1989 (Hsu and Cheng 2018). Another protein from the fruiting bodies of *G. lucidum* is ganodermin, which has a molecular mass of 15 kDa and has antifungal activity.

## Health benefits of *G. lucidum*

### Antioxidant effects

Free radicals are unstable and highly reactive chemical entities which contain one or more unpaired electrons and can be uncharged or charged. Free radicals are beneficial to the cell signalling and immune system, as well as maintenance of normal body functioning. However, excessive formation and/or insufficient removal of reactive oxygen species (ROS) and reactive nitrogen species (RNS), known as ‘oxidative stress’, may modulate the blood vessel wall, creating an environment that facilitates the progression of atherosclerosis, and leading to various illnesses, such as heart disease, diabetes and cancer (Johansen et al. 2005; Ullah et al. 2016).

*In vitro* studies demonstrated that several constituents of *G. lucidum*, in particular triterpenoids and polysaccharides, exhibit antioxidant activity, reducing power, scavenging and chelating abilities (Mau et al. 2002; Saltarelli et al. 2009; Wu and Wang 2009; Liu et al. 2010; Sarmadi and Ismail 2010; Kozarski et al. 2011; Ferreira et al. 2015; Krishna et al. 2016). In contrast, polysaccharide extracts of *G. lucidum* have superoxide and hydroxyl radical scavenging activities but do not have antioxidative activity as measured by detecting malondialdehyde (MDA) contents of liver microsomes (Liu et al. 1997). It has been demonstrated that the phenolic compounds from the fresh fruiting bodies of *G. lucidum* exhibit strong 1,1-diphenyl-2-picrylhydrazyl (DPPH) radical scavenging activity but low superoxide dismutase (SOD) activity. The study also showed that DPPH radical scavenging activity and SOD activity were positively correlated with phenolic compounds including caffeic acid, catechin, ferulic acid, gallic acid, myricetin, naringin, pyrogallol, protocatechuic acid, homogentisic acid, and quercetin, as well as total phenolic compounds (Kim et al. 2008). A study comparing the antioxidant activities of four of the most widely known mushrooms, including *G. lucidum*, demonstrated that polysaccharide extracts exhibited a strong correlation between the reducing power and the total amount of phenols and α-glucans, while a correlation between the reducing power and the amount of total polysaccharides and proteins was not found (Kozarski et al. 2012).

*In vivo* studies have shown that *G. lucidum* increases the activity of the antioxidant enzymes SOD and catalase (CAT), which are involved in removing harmful ROS (Cherian et al. 2009; Yurkiv et al. 2015; Vitak et al. 2017; Rahman et al. 2018). In an ischaemia and reperfusion isolated perfused rat heart model, administration of *G. lucidum* extract (400 mg/kg for 15 days) exhibited antioxidant properties and the author concluded that the cardioprotective properties of *G. lucidum* extract are related to its antioxidant effects (Lasukova et al. 2015). A study in rats showed that *G. lucidum* ethanol extract (250 mg/kg body weight) ameliorated the cardiotoxicity of adriamycin by reducing the increase in lipid peroxidation and reversing the decrease in the antioxidant enzymes, glutathione peroxidase (GPx), glutathione-*S*-transferase (GST), SOD and CAT in the heart tissue (Rajasekaran and Kalaimagal 2012). The cardioprotective effect of *G. lucidum* may be attributed to the antioxidant chemicals triterpenes and polysaccharides (Wachtel-Galor et al. 2004b). In a carotid-artery-ligation mouse model, daily oral *G. lucidum* (300 mg/kg/day) prevented neointimal thickening 2 weeks after ligation. Furthermore, subcutaneous injections of ganoderma triterpenoid (GT) crude extract (300 mg/kg/day) abolished ligation-induced neointima formation. The authors concluded that GTs prevent atherogenesis by eliminating disturbed flow-induced oxidative stress through inhibiting the induction of a series of atherogenic factors, as well as inflammation (Hsu et al. 2018).

A short-term supplementation study over 10 days in healthy subjects showed an improvement in antioxidant status (Wachtel-Galor et al. 2004a), but a longer double-blind, placebo-controlled, cross-over intervention study over 4 weeks with a commercially available encapsulated Lingzhi preparation (1.44 g Lingzhi/day; equivalent to 13.2 g fresh mushroom/day) showed no significant effects in a range of biomarkers for antioxidant status, cardiovascular risk, DNA damage, immune status, and inflammation (Wachtel-Galor et al. 2004b). A placebo-controlled cross-over study in 42 healthy subjects examined the antioxidation and hepatoprotective efficacy of triterpenoids and polysaccharide-enriched *G. lucidum*, which was taken as a 225 mg capsule containing 7% triterpenoid-ganoderic acid (A, B, C, C5, C6, D, E and G), 6% polysaccharide peptides with a few essential amino acids and trace elements, once daily for 6 consecutive months (Chiu et al. 2017). The treatment showed an improvement in total antioxidant capacity, total thiols and glutathione content in plasma, significantly enhanced activities of antioxidant enzymes (SOD, CAT, GPx and glucose-6-phosphate dehydrogenase), and reduced the levels of thiobarbituric acid reactive substances, 8-hydroxy-deoxy-guanosine and hepatic marker enzymes, glutamic-oxaloacetic transaminase and glutamic-pyruvic transaminase. Mild fatty liver detected by abdominal ultrasonic examination was reversed to normal with *G. lucidum* treatment.

### Hypoglycaemic activity

Hyperglycaemia may increase the susceptibility to lipid peroxidation and modulate glucose metabolism in the body, which ultimately contributes to the increased incidence of atherosclerosis or further accelerates its progression (Giugliano et al. 1996; Poznyak et al. 2020). Insulin treatment is essential for people with type 1 diabetes. In type 2 diabetes mellitus (T2DM), lifestyle modification is recommended. If lifestyle modification is not sufficient in achieving glycemic control, patients should be treated initially with metformin (American Diabetes Association 2020). Metformin belongs to the biguanide class of drugs, which originate from the plant goat’s rue or French lilac (*Galega officinalis*, Linnaeus, [Fabaceae]) (Witters 2001). Recently, the glucagon-like peptide 1 (GLP-1) receptor agonists and sodium-glucose cotransporter 2 (SGLT2) inhibitors, which were developed from phlorizin, a natural compound isolated from the bark of apple roots (Tomlinson et al. 2017), have been considered suitable for first-line treatment in some patients with T2DM who have concomitant cardiac or renal disease, in order to improve cardiovascular outcome benefits (Davies et al. 2018).

The hypoglycaemic effects of various extracts from *G. lucidum* have been studied in different animal models of diabetes and in *in vitro* experiments to identify mechanisms (Ma et al. 2015; Wang et al. 2016; Winska et al. 2019). The main *in vitro*, animal and clinical studies investigating the hypoglycaemic effects of *G. lucidum* are summarised in Tables 1–3, respectively.

**Table 1.** *In vitro* studies on the hypoglycaemic effects of *G. lucidum*.

References	Model	Interventions	Findings
Zhang et al. 2003	Alloxan-induced pancreatic islet damage	*Gl*-PS polysaccharides from the fruiting body of *G. lucidum*	*Gl*-PS showed a protective effect
Fatmawati et al. 2009	Human aldose reductase activity	Methanol extracts of 17 medicinal and edible mushrooms	*G. lucidum* showed the highest aldose reductase inhibitory activity
Fatmawati et al. 2010	Human aldose reductase activity	Ganoderic acid Df isolated from the fruiting body of *G. lucidum*	Ganoderic acid Df showed potent human aldose reductase inhibitory activity
Fatmawati et al. 2011a	Human α-glucosidase activity	Chloroform extract of the fruiting body of *G. lucidum*	Ganoderol B identified as an active α-glucosidase inhibitor
Pan et al. 2015	PTP1B activity	FYGL proteoglycan isolated from *G. lucidum*	Competitive inhibitor of PTP1B
Yang et al. 2018a	Liver tissues of ob/ob mice and HepG2 cells	FYGL proteoglycan isolated from *G. lucidum*	Inhibited PTP1B overexpression, improved IRS1 phosphorylation, activated PI3K/Akt cascades, increased phosphorylation of GSK3β, enhanced insulin-stimulated glycogen synthesis
Yang et al. 2018b	Rat myoblast L6 cells	FYGL proteoglycan isolated from *G. lucidum*	Increased insulin-stimulated glucose uptake, inhibited PTP1B expression, increased IRS1 phosphorylation, activated PI3K/Akt, increased phosphorylation of AMPK and up-regulated expression of GLUT4

Akt: protein kinase B; AMPK: adenosine monophosphate-activated protein kinase; FYGL: Fudan-Yueyang *Ganoderma lucidum*; GLUT4: glucose transporter type 4; GSK3β: glycogen synthase kinase-3β; IRS1: insulin receptor substrate 1; PI3K: phosphatidylinositol-3 kinase; PTP1B: protein tyrosine phosphatase 1B.

**Table 2.** Animal *s*tudies on the hypoglycaemic effects of *G. lucidum*.

References	Animal model	Interventions	Findings
Hikino et al. 1985	Normal and alloxan-induced hyperglycaemic mice	Water extracts (10^4^ mg/kg crude drug equivalent, i.p.) of the fruiting bodies of *G. lucidum* for 7 or 27 h	Reduced plasma glucose and 2 glycans, ganoderans A and B, with hypoglycaemic action isolated
Hikino et al. 1989	Normal and glucose-loaded mice	Ganoderan B	Increased insulin and altered enzyme activities
Kino et al. 1990	Autoimmune diabetes model in non-obese mice	Ling Zhi-8 immunomodulatory protein (10.3 − 12.6 mg/kg twice weekly) from 4 weeks of age, followed up to 42 weeks of age	Prevented development of autoimmune diabetes by immunosuppressive mechanism
Zhang et al. 2003	Alloxan-induced diabetic mice	Pre-treatment with intragastric *Gl*-PS (50 − 200 mg/kg) for 10 days	*Gl*-PS partly protected beta cells from necrosis
Zhang & Lin 2004	Normal fasted mice	*Gl*-PS (25 − 100 mg/kg) given by single intraperitoneal injections	Reduced serum glucose and increased insulin levels
He et al. 2006	Streptozotocin-induced diabetic mice	*Gl*-PS (125 and 250 mg/kg) given for 8 weeks	Reduced serum glucose, increased insulin levels and delayed progression of diabetic renal disease
Seto et al. 2009	Genetically obese/diabetic (+db/+db) and lean (+db/+m) mice	Water extract of *G. lucidum* (0.003, 0.03 and 0.3 g/kg) for 4 weeks, oral gavage	Extract reduced serum glucose and liver PEPCK expression
Li et al. 2011	Streptozotocin-induced diabetic mice	*Gl*-PS at low (50 mg/kg) and high (150 mg/kg) dose for 28 days	Reduced serum glucose, increased insulin levels and improvements in blood lipids
Teng et al. 2011	Streptozotocin-induced diabetic mice	FYGL proteoglycan from *G. lucidum* (50 and 150 mg/kg, oral dose) for up to 4 weeks	Reduced plasma glucose with effect comparable with metformin
Teng et al. 2012	Streptozotocin-induced diabetic rats	FYGL proteoglycan from *G. lucidum* (40 and 120 mg/kg, oral dose) for 30 days	Reduced plasma glucose, increased insulin and inhibited PTP1B
Zheng et al. 2012	Streptozotocin-induced diabetic rats	Low-molecular-weight *Gl*-PS (200 mg/kg) orally for 8 weeks	Reduced serum glucose appeared related to protection of pancreatic β-cells
Xiao et al. 2012	Streptozotocin-induced diabetic mice	Polysaccharides from *G. lucidum* (50 or 100 mg/kg/day) given for 7 days	Reduced fasting serum glucose and insulin levels
Pan et al. 2013	Obese/diabetic (+db/+db) mice	FYGL proteoglycan from *G. lucidum* (75, 250, or 450 mg/kg) for 8 weeks	Reduced HbA1c, increased insulin and C-peptide levels, increased glucokinase and lowered PEPCK activities
Sarker 2015	Rats with alloxan- or corticosteroid-induced diabetes	A petroleum ether extract and a methanol extract of *G. lucidum* (200, 400, 600 and 800 mg/kg/day) for 7 days	Reduced fasting and postprandial plasma glucose and HbA1c, increased plasma insulin levels and improved lipid profile
Xiao et al. 2017	Streptozotocin-induced diabetic mice	F31 polysaccharide from *G. lucidum* (50 mg/kg/day)	Decreased fasting serum glucose, fasting serum insulin and liver glucose regulatory enzymes
Ratnaningtyas et al. 2018	Alloxan-induced diabetic rats	Ethanol extract of *G. lucidum* powdered fruiting bodies (250, 500 and 1000 mg/kg) for 14 days	Dose-dependent reduction in blood glucose, reduction in HbA1c, and increase in insulin
Bach et al. 2018	Streptozotocin-induced diabetic rats	Hydroethanolic extract of *G. lucidum* (1 mL/kg/day) for 30 days	Reduced plasma glucose and lipid levels

FYGL: Fudan-Yueyang *Ganoderma lucidum*; HbA1c: Glycosylated Haemoglobin Level; *Gl*-PS: *Ganoderma lucidum* polysaccharides; PEPCK: phosphoenolpyruvate carboxykinase; PTP1B: protein tyrosine phosphatase.

**Table 3.** Human *s*tudies on the hypoglycaemic effects of *G. lucidum*.

References	Subjects	Interventions	Findings
Gao et al. 2004b	62 patients with T2DM	Multi-centered randomised controlled trial of Ganopoly^TM^ 1800 mg 3 times daily versus placebo for 12 weeks	Reduced HbA1c and fasting and postprandial plasma glucose levels with Ganopoly^TM^
Wang et al. 2008	46 patients with T2DM	Randomised, double-blind, placebo-controlled dry extract of *G. lucidum* 3000 mg or placebo for 12 weeks	No changes in fasting glucose or HbA1c but the plasma glucose area under the curve during a meal tolerance test was reduced more with *G. lucidum* extract
Chu et al. 2012	23 subjects with borderline elevations of blood pressure and/or cholesterol	Randomised, double-blind, cross-over study with a Lingzhi product 1.44 g daily or placebo for 12 weeks	No significant effect on HbA1c, fasting plasma glucose, blood pressure or lipids. Plasma insulin and HOMA-IR reduced with Lingzhi compared to placebo
Klupp et al. 2016	84 patients with T2DM and metabolic syndrome	Randomised controlled trial of *G. lucidum* 3 g/day or *G. lucidum* plus *Cordyceps sinensis* capsules, versus placebo for 16 weeks	No significant effect on HbA1c, fasting plasma glucose, blood pressure or lipids

HbA1c: Glycosylated Haemoglobin Level; HOMA-IR: homeostasis model assessment-insulin resistance; T2DM: type 2 diabetes mellitus.

#### Hypoglycaemic activity of triterpenoids

**Figure 1.**
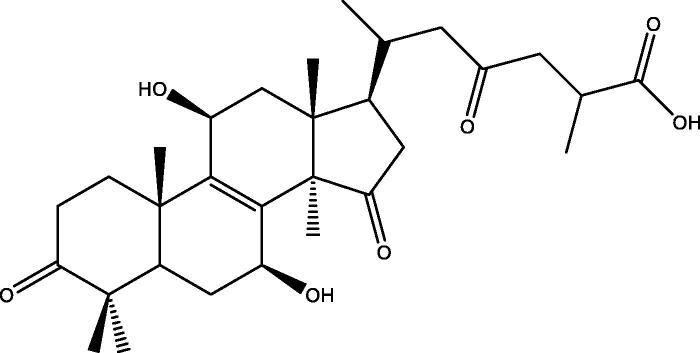
Chemical structure of ganoderic acid Df. The hydroxyl group at C-11 and the carbonyl group at C-15 along with the hydroxyl group at C-7 are thought to be important for inhibition of aldose reductase (Fatmawati et al. 2010, 2011b).

**Figure 2.**
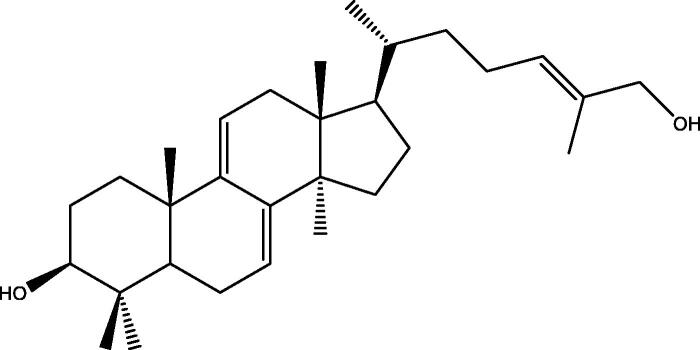
Chemical structure of ganoderol B. The hydroxyl group at C-3 and the double-bond in the side chain are thought to be important for α-glucosidase inhibitory activity (Fatmawati et al. 2013).

A series of *in vitro* studies by Fatmawati and colleagues have identified that methanol extract from the fruiting bodies of *G. lucidum* has a strong inhibitory effect on human aldose reductase activity. Ganoderic acid Df (Figure 1), a lanostane-type triterpenoid, exhibited potent aldose reductase inhibitory activity with an IC_50_ value of 22.8 µM (Fatmawati et al. 2009, 2010). Fatmawati et al. (2011a) subsequently demonstrated that ganoderol B (Figure 2), which was isolated from a chloroform extract of *G. lucidum*, was effective in inhibiting α-glucosidase activity with an IC_50_ value of 119.8 µM and the inhibitory effect was stronger than that of acarbose, which is commonly used as a medication to inhibit α-glucosidase in patients with T2DM. Structure-activity studies were performed to identify the structural requirements of lanostane-type triterpenoids from *G. lucidum*, which were necessary to increase α-glucosidase inhibitory activity (Fatmawati et al. 2013).

#### Hypoglycaemic activity of proteoglycans/peptidoglycans

Inhibition of PTP1B activity has been regarded as a potential therapy for T2DM for many years (Johnson et al. 2002). Fudan-Yueyang-*G. lucidum* (FYGL), which is a water soluble macromolecular proteoglycan extracted from the fruiting bodies of *G. lucidum*, inhibits PTP1B activity with an IC_50_ value of 5.12 ± 0.05 µg/mL (Teng et al. 2011). FYGL enhances glycogen synthesis and inhibits the expression of glycogen synthase kinase-3β (GSK3β) in liver tissues of ob/ob mice and HepG2 cells probably via modulating insulin receptor substrate 1 (IRS1)/phosphatidylinositol-3 kinase (PI3K)/protein kinase B (Akt)/AMP-activated protein kinase (AMPK)/GSK3β cascades (Yang et al. 2018a). In rat myoblast PTP1B-transfected L6 cells, FYGL improves insulin resistance by regulating IRS1-glucose transporter type 4 (GLUT4) cascades in the insulin signalling pathway (Yang et al. 2018b). In streptozotocin-induced T2DM mice, FYGL reduces plasma glucose levels with an effect comparable with metformin and rosiglitazone, via inhibiting the PTP1B expression and activity, and consequently modulating the tyrosine phosphorylation level of the insulin receptor (IR) 13-subunit (Teng et al. 2011, 2012). In addition, FYGL improves the plasma biochemistry indexes associated with T2DM-accompanied metabolic disorders, including free fatty acids, triglycerides (TG), total cholesterol (TC), low-density lipoprotein cholesterol (LDL-C), and high-density lipoprotein cholesterol (HDL-C) (Teng et al. 2012). Further mechanistic studies in db/db mice found that the hypoglycaemic effect of FYGL is associated with its ability to enhance insulin secretion, decrease hepatic glucose output, and increase adipose and skeletal muscle glucose disposal (Pan et al. 2013, 2014). In normal and alloxan-induced hyperglycaemic mice, water extraction yielded from the fruiting bodies of *G. lucidum* and the two peptidoglycans, ganoderans A and B, subsequently produced through fractionation have all shown hypoglycaemic activity (Hikino et al. 1985). Administration of ganoderan B increases plasma insulin levels in normal and glucose-loaded mice; it also increases the activities of hepatic glucokinase, phosphofructokinase and glucose-6-phosphate dehydrogenase, decreases hepatic glucose-6-phosphatase (G6Pase) and glycogen synthetase activities and does not affect the activities of hexokinase and glycogen phosphorylase (GP) (Hikino et al. 1989).

#### Hypoglycaemic activity of *Ganoderma* polysaccharides

Hypoglycaemic effects of polysaccharides from *G. lucidum* (*Gl*-PS) have been demonstrated in several *in vitro* and *in vivo* studies. *Gl*-PS showed a protective effect against alloxan-induced damage to pancreatic islets *in vitro*. Pre-treatment with intragastric *Gl*-PS (50-200 mg/kg) for 10 days produced hypoglycaemic effects via its scavenging ability to protect the pancreatic β-cells from alloxan-induced necrosis (Zhang et al. 2003). *Gl*-PS (25-100 mg/kg) given by single intraperitoneal injections to normal fasted mice reduced serum glucose levels after 3 and 6 h in a dose-dependent manner and increased insulin levels from 1 h after administration via enhancing Ca^2+^ influx into pancreatic β cells (Zhang and Lin 2004). Furthermore, administration of *Gl*-PS produced hypoglycaemic effects and an improvement in lipid profile in streptozotocin-induced diabetic mice (He et al. 2006; Li et al. 2011; Zheng et al. 2012). It has been suggested that the hypoglycaemic effect is mainly through preventing apoptosis of pancreatic β-cells and enhancing β-cells regeneration (Zheng et al. 2012), and a modulation of serum insulin and hepatic mRNA levels of several key enzymes involved in gluconeogenesis and/or glycogenolysis, including GP, fructose-1,6-bisphosphatase (FBPase), phosphoenolpyruvate carboxykinase (PEPCK), and G6Pase (Xiao et al. 2012). Xiao et al. (2017) isolated F31, a β-heteropolysaccharide with a weight-average molecular weight of 15.9 kDa, from *Gl*-PS. The mechanism of action of *Gl*-PS F31 may be associated with down-regulation of the hepatic glucose regulated enzyme mRNA levels via AMPK activation, improvement of insulin resistance, and reduction of epididymal fat/body weight ratio (Xiao et al. 2017). An integrative analysis of transcriptomics and proteomics data from the liver from F31-treated diabetic db/db mice found that genes in the glycolysis and gluconeogenesis pathways, insulin pathway, and lipid metabolism pathways showed significantly different expression compared to the untreated mice and that microRNAs probably participated in the regulation of the genes involved in glucose metabolism (Xiao et al. 2018).

#### Hypoglycaemic activity of *Ganoderma* extracts

Some other studies used extracts of *G. lucidum* in which the active constituents were not clearly identified. A water-extract of *G. lucidum* given to lean (+db/+m) and genetically obese/diabetic (+db/+db) mice lowered the serum glucose level in + db/+db mice after one week of treatment and in + db/+m mice after 4 weeks, through the down-regulation of the hepatic PEPCK gene expression (Seto et al. 2009). A study in alloxan-and steroid-induced diabetic rats showed that a petroleum ether extract and a methanol extract of *G. lucidum* given orally at 200, 400, 600 and 800 mg/kg/day for 7 days reduced plasma glucose levels, increased insulin sensitivity, and decreased lipid levels, and the suspected bioactive chemicals were polysaccharides available in the extracts (Sarker 2015). A hypoglycaemic effect was also observed following administration of an alcoholic extract of *G. lucidum* (250, 500, and 1000 mg/kg) given for 14 days in alloxan-induced diabetic rats (Ratnaningtyas et al. 2018). Another recent study in streptozotocin-induced diabetic rats showed that a hydroethanolic extract of *G. lucidum* containing β-glucan, proteins, and phenols, reduced plasma glucose and lipid levels through preservation of pancreatic islets (Bach et al. 2018).

#### Hypoglycaemic activity of *Ganoderma* proteins

Ling Zhi-8 (LZ-8), an immunomodulatory protein isolated from the mycelial extract of *G. lucidum*, prevented the development of autoimmune diabetes by reducing antigen-induced antibody formation in non-obese diabetic mice (Kino et al. 1990). In a model of transplanted allogeneic pancreatic rat islets, LZ-8 delayed the rejection process of allografted islets (van der Hem et al. 1995).

#### Evidence from clinical studies

Clinical studies of the hypoglycaemic/antidiabetic effects of *G. lucidum* products are very limited. In a placebo-controlled study in 62 patients with T2DM, administration of Ganopoly™ at 1800 mg three times daily for 12 weeks reduced fasting and postprandial plasma glucose levels, as well as HbA1c (Gao et al. 2004b). Administration of a dry extract of *G. lucidum* (3 g) in addition to regular oral hypoglycaemic agents for 12 weeks did not affect fasting glucose or HbA1c; however, the plasma glucose area under the curve during a meal tolerance test was reduced more significantly in patients taking *G. lucidum* (Wang et al. 2008). A randomised, double-blind, placebo-controlled, cross-over study with placebo-controlled run-in and cross-over periods of a Lingzhi product at a dose of 1.44 g daily for 12 weeks was performed in subjects with borderline elevations of blood pressure and/or cholesterol. There were reductions in plasma insulin and homeostasis model assessment-insulin resistance with Lingzhi compared to placebo. The subjects in this study had normal plasma glucose levels and it was speculated that the effects on insulin and insulin resistance would be greater in subjects with impaired glucose tolerance or T2DM (Chu et al. 2012). However, in a more recent study in 84 patients with T2DM and metabolic syndrome, administration of *G. lucidum* alone or combined with *Cordyceps sinensis* [now called *Ophiocordyceps sinensis* (Berk.) Sacc. (Ophiocordycipitaceae)], over 16 weeks, did not show any improvement in hyperglycaemia and cardiovascular risk factors (Klupp et al. 2016). It is noteworthy that different extracts of *G. lucidum* will have different components, therefore it may not be appropriate to compare the results from different studies.

### Effects on dyslipidaemia

Dyslipidaemia which is characterised by decreased levels of HDL-C and accompanied with increased levels of TG, apo B, and small dense LDL particles, is an important modifiable risk factor for the development of atherosclerosis and CVD. Guidelines for the treatment of lipid disorders recommend initiating treatment with the 3-hydroxy-3-methylglutaryl-coenzyme A (HMG-CoA) reductase inhibitors or statins (Grundy et al. 2019; Mach et al. 2020). Statins have their origin in products isolated from fungi (Endo 2004).

**Figure 3.**
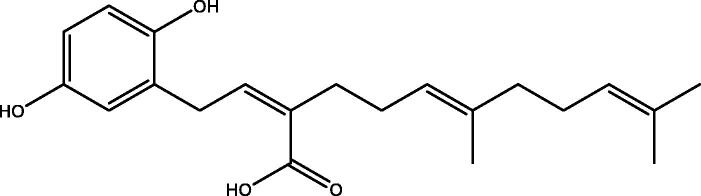
Chemical structure of ganomycin B. Ganomycin B showed strong inhibitory activity against HMG-CoA reductase with an IC_50_ of 14.3 μM (Chen et al. 2017).

*In vitro* studies showed that polysaccharides and oxygenated triterpenoids from *G. lucidum* have a very broad spectrum of biological activities and pharmacological effects. Some types of ganoderic acid might reduce cholesterol by inhibiting HMG-CoA reductase, like the statin drugs (Shiao 2003). Compounds isolated from fruiting bodies of *G. lucidum* including ganolucidic acid eta, ganoderenic acid K, and the farnesyl hydroquinones (ganomycin J and ganomycin B), showed strong inhibitory activity against HMG-CoA reductase (Figure 3) (Chen et al. 2017).

The cholesterol-lowering properties of *G. lucidum* have been demonstrated in a series of *in vitro* and *ex vivo* studies, and in hamsters and minipigs (Berger et al. 2004). The organic fractions containing oxygenated lanosterol derivatives inhibited cholesterol synthesis in T9A4 hepatocytes. The investigators found that both 2.5 and 5% dried *G. lucidum* reduced hepatic microsomal *ex-vivo* HMG-CoA reductase activity. In hamsters, administration of 5.0% dried *G. lucidum* decreased TC and HDL-C but not LDL-C, whereas in minipigs, 2.5% dried *G. lucidum* reduced all these parameters.

The improvements in the lipid profile in some diabetic animal models and in patients with T2DM treated with *G. lucidum* products may be related to the improvement in glycemic control, rather than a direct effect on lipid metabolism as hyperglycaemia is often associated with elevated TG and reduced HDL-C (Taskinen and Borén 2015). In a randomised, double-blind, cross-over study in 26 patients with borderline elevations of blood pressure and/or cholesterol, administration of Lingzhi (1.44 g extract/d) for 12 weeks produced a non-significant trend for reduction in TG and increase in HDL-C (Chu et al. 2012). Those changes could have been related to improvements in insulin resistance as these lipid abnormalities, hypertension, central obesity and insulin resistance cluster together in the metabolic syndrome.

### Antihypertensive effects

The most recent guidelines for the management of hypertension recommend initiating antihypertensive drug therapy in most patients with a combination of two different drugs from the classes of thiazide diuretics, calcium channel blockers, angiotensin converting enzyme (ACE) inhibitors, or angiotensin receptor blocker (ARBs) (Whelton et al. 2018; Williams et al. 2018).

Triterpenes and *G. lucidum* proteins have been demonstrated to possess potent ACE-inhibitory properties *in vitro* (Abdullah et al. 2012; Mohamad Ansor et al. 2013). Mohamad Ansor et al. (2013) reported that the protein fractions from the mycelia of *G. lucidum* contain highly potent anti-ACE proteins with IC_50_ values below 200 μg/mL. Furthermore, three small peptides with ACE-inhibitory activity, including Gln-Leu-Val-Pro (QLVP), Gln-Asp-Val-Leu (QDVL), and Gln-Leu-Asp-Leu (QLDL), were recently isolated from *G. lucidum* mycelia (Wu et al. 2019). Notably, QLVP worked in a mixed-type manner against ACE and has an IC_50_ value of 127.9 µmol/L.

A transverse aortic constriction (TAC) mouse model of pressure overload-induced cardiomyopathy and heart failure revealed that administration of oral *Ganoderma* spore oil every other day for 14 days normalised ejection fraction, corrected the fractional shortening and reduced left ventricular hypertrophy. The cardioprotective effect is associated with reduced expression of circular RNA circ-Foxo3, which plays a role in the pathogenesis of heart failure (Xie et al. 2016).

An early uncontrolled trial in Japanese showed that supplementation with *G. lucidum* extract (240 mg daily) for 6 months reduced blood pressure in hypertensive patients but not borderline hypertensive or normotensive patients (Kanmatsuse et al. 1985). In a double-blind, randomised, placebo-controlled study in 160 patients with confirmed coronary heart disease (CHD), treatment with *G. lucidum* polysaccharides (Ganopoly™) for 12 weeks improved the symptoms of CHD and reduced average blood pressure from 142.5/96.4 mmHg to 135.1/92.8 mmHg, whereas there was no significant blood pressure reduction in the control group (Gao et al. 2004a). Serum TC also decreased significantly with Ganopoly™ therapy, but not in the control group.

### Anti-inflammatory effects

Inflammation is a physiological response to harmful stimuli that are physical, chemical, or biological in nature. A number of inflammatory markers, such as high-sensitivity C-reactive protein (hsCRP), interleukin (IL)-6, IL-1, and tumour necrosis factor (TNF)-α, have been shown to be associated with obesity, metabolic syndrome, and an elevated risk of chronic diseases (Pravenec et al. 2011; Dallmeier et al. 2012). Elevated circulating levels of hsCRP and IL-6 predict the development of T2DM through diminishing insulin sensitivity (Guarner & Rubio-Ruiz 2015). Obesity-induced inflammation has been implicated as a risk factor in the pathogenesis of T2DM, insulin resistance, CVD, and metabolic syndrome (Kumar et al. 2019).

There are several *in vitro* studies showing the anti-inflammatory effect of *G. lucidum* extracts. The triterpene extract from *G. lucidum* reduced the secretion of TNF-α and IL-6, and inflammatory mediator nitric oxide (NO) and prostaglandin E(2) (PGE2) from lipopolysaccharide (LPS)-activated murine macrophages via inhibition of nuclear factor-κB (NF-κB) and activator protein 1 (AP-1) signalling (Dudhgaonkar et al. 2009). *G. lucidum* sterols downregulated the mRNA expressions of NO, TNF-α, IL-1β, and IL-6, and attenuated LPS-induced cell polarisation by modulating mitogen-activated protein kinase (MAPK) and NF-κB pathways (Xu et al. 2021). Furthermore, *G. lucidum* ethanol extract reduced the excessive production of NO, PGE2, and pro-inflammatory cytokines, IL-1β, and TNF-α via inhibition of the NF-κB and toll-like receptor signalling pathways in LPS-stimulated BV2 microglial cells (Yoon et al. 2013).

In an *in vivo* study, administration of water extract of *G. lucidum* (2 g/kg, s.c.) 1 h prior to applying carrageenan reduced both the first and second phases of carrageenan-induced inflammation (Lin et al. 1993). It has been demonstrated that both ethyl acetate and 70% methanol extracts of *G. lucidum* (500 and 1000 mg/kg) produced anti-inflammatory effects against carrageenan-induced acute and formalin-induced chronic inflammation in mice and the effect was comparable to that of the standard reference drug, diclofenac (10 mg/kg) (Sheena et al. 2003).

**Figure 4.**
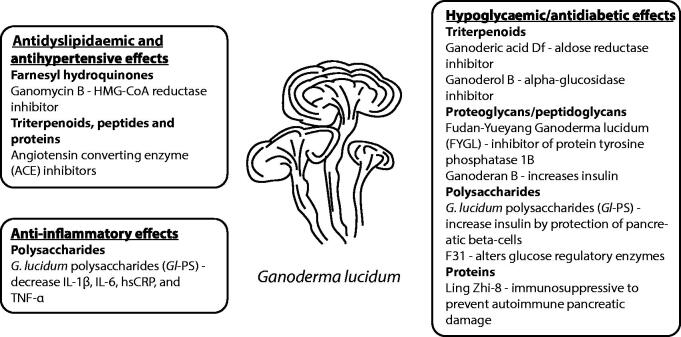
Potential mechanisms for cardiovascular disease prevention and therapy with constituents of *Ganoderma lucidum*.

The anti-inflammatory effect of *G. lucidum* supplementation has been investigated in several small scale trials. In a clinical trial involving 45 ST-elevation myocardial infarction (STEMI) and non-STEMI patients, the polysaccharides of *G. lucidum* (750 mg/day in 3 divided doses for 90 days) decreased the levels of IL-1 and TNF-α, as well as the MDA levels (Sargowo et al. 2019). In a recent randomised closed-label clinical trial involving 38 patients with atrial fibrillation, consumption of polysaccharides of *G. lucidum* (PT Sahabat Lingkungan Hidup, Surabaya, Indonesia), 3 times a day for 90 days, reduced significantly the systolic and diastolic blood pressure, heart rate, LDL-C, IL-1β, IL-6, hsCRP, and TNF-α, compared to placebo-treated patients (Rizal et al. 2020). These data suggest that *G. lucidum* polysaccharide peptides may have beneficial effects against factors involved in the pathogenesis of atherosclerosis and atrial fibrillation. The main active compounds which have been shown to influence some of the major risk factors for CVD are shown in Figure 4.

## Adverse effects

*G. lucidum* is generally regarded as safe and is listed in the safest drug class (Class 1 Drug) in the *American Herbal Products Association Botanical Safety Handbook* with no known herb-drug interactions (McGuffin et al. 1997). Recent human clinical trials with *G. lucidum* have included laboratory safety parameters such as hepatic, renal, and hematological biomarkers and no pathological abnormality or serious adverse event has been reported (Klupp et al. 2015, 2016). Mild symptomatic adverse effects such as dry mouth, sore throat, and nausea have been reported occasionally. A case of hepatotoxicity related to *G. lucidum* mushroom powder was reported from Hong Kong in 2004, but this was thought to be due to the excipient ingredients (Yuen et al. 2004). Another case of fatal fulminant hepatitis in a patient taking Lingzhi in powder form was reported from Thailand in 2007 (Wanmuang et al. 2007). Such cases do need careful assessment before attributing the effects to *G. lucidum* components, but they also illustrate the need to be vigilant with herbal treatments.

It is important to be cautious when taking herbal supplements in combination with conventional medications, particularly those that are very sensitive to herb or drug interactions such as warfarin. Most herbal supplements are contraindicated in patients taking warfarin. *G. lucidum* may have a mild antithrombotic effect itself in high doses and this could increase the effect of other anticoagulant or antiplatelet medications, including aspirin (Kumaran et al. 2011), resulting in an increased risk of bruising or bleeding. In patients taking other prescription medications, it is generally better to separate the intake of those medications and *G. lucidum* products by at least two hours in case there is any interference with drug absorption.

## Conclusions

*G. lucidum* has a reputation for many beneficial effects from a historical perspective and its safety has largely been established by empirical observation. The beneficial effects are supported by several *in vitro* studies and studies in animals, but clinical trials in humans in the cardiovascular field are limited. Secondly, the use of different products in the clinical trials makes it difficult to compare the results. In the prevention and treatment of CVD, the hypoglycaemic effects of *G. lucidum* are the best established properties from the *in vitro* and animal studies, but these benefits have not been confirmed in recent clinical trials. Components from *G. lucidum* herbal materials have been identified with lipid-lowering and antihypertensive effects and compounds with specific mechanisms of action have been isolated. Nevertheless, the content of these components and their bioavailability in different *G. lucidum* formulations are uncertain and clinical trials in these areas have been inadequate. Further studies are needed to isolate all the active ingredients with known biological activity, and to characterise their bioavailability for specific indications before clinical trials pertaining to the use of *G. lucidum* products for relevant clinical benefits are conducted. Clinical trials should be performed in subjects with abnormal baseline levels of cardiovascular risk factors that are being targeted so that improvements can be seen more readily.
